# Investigating GABA Neuron–Specific Androgen Receptor Knockout in two Hyperandrogenic Models of PCOS

**DOI:** 10.1210/endocr/bqae060

**Published:** 2024-05-24

**Authors:** Irene E Sucquart, Chris Coyle, Valentina Rodriguez Paris, Melanie Prescott, Kelly A Glendining, Kyoko Potapov, Denovan P Begg, Robert B Gilchrist, Kirsty A Walters, Rebecca E Campbell

**Affiliations:** Fertility & Research Centre, School of Clinical Medicine, University of New South Wales Sydney, Randwick, NSW 2031, Australia; Centre of Neuroendocrinology and Department of Physiology, School of Biomedical Sciences, University of Otago, Dunedin, New Zealand 9054; Fertility & Research Centre, School of Clinical Medicine, University of New South Wales Sydney, Randwick, NSW 2031, Australia; School of Biomedical Sciences, University of New South Wales Sydney, Randwick, NSW 2052, Australia; Centre of Neuroendocrinology and Department of Physiology, School of Biomedical Sciences, University of Otago, Dunedin, New Zealand 9054; Centre of Neuroendocrinology and Department of Physiology, School of Biomedical Sciences, University of Otago, Dunedin, New Zealand 9054; Centre of Neuroendocrinology and Department of Physiology, School of Biomedical Sciences, University of Otago, Dunedin, New Zealand 9054; Department of Behavioural Neuroscience, School of Psychology, University of New South Wales Sydney, Randwick, NSW, Australia; Fertility & Research Centre, School of Clinical Medicine, University of New South Wales Sydney, Randwick, NSW 2031, Australia; Fertility & Research Centre, School of Clinical Medicine, University of New South Wales Sydney, Randwick, NSW 2031, Australia; Centre of Neuroendocrinology and Department of Physiology, School of Biomedical Sciences, University of Otago, Dunedin, New Zealand 9054

**Keywords:** androgen receptor, polycystic ovary syndrome, mice, DHT, GABA

## Abstract

Androgen excess is a hallmark feature of polycystic ovary syndrome (PCOS), the most common form of anovulatory infertility. Clinical and preclinical evidence links developmental or chronic exposure to hyperandrogenism with programming and evoking the reproductive and metabolic traits of PCOS. While critical androgen targets remain to be determined, central GABAergic neurons are postulated to be involved. Here, we tested the hypothesis that androgen signaling in GABAergic neurons is critical in PCOS pathogenesis in 2 well-characterized hyperandrogenic mouse models of PCOS. Using cre-lox transgenics, GABA-specific androgen receptor knockout (GABARKO) mice were generated and exposed to either acute prenatal androgen excess (PNA) or chronic peripubertal androgen excess (PPA). Females were phenotyped for reproductive and metabolic features associated with each model and brains of PNA mice were assessed for elevated GABAergic input to gonadotropin-releasing hormone (GnRH) neurons. Reproductive and metabolic dysfunction induced by PPA, including acyclicity, absence of corpora lutea, obesity, adipocyte hypertrophy, and impaired glucose homeostasis, was not different between GABARKO and wild-type (WT) mice. In PNA mice, acyclicity remained in GABARKO mice while ovarian morphology and luteinizing hormone secretion was not significantly impacted by PNA or genotype. However, PNA predictably increased the density of putative GABAergic synapses to GnRH neurons in adult WT mice, and this PNA-induced plasticity was absent in GABARKO mice. Together, these findings suggest that while direct androgen signaling in GABA neurons is largely not required for the development of PCOS-like traits in androgenized models of PCOS, developmental programming of GnRH neuron innervation is dependent upon androgen signaling in GABA neurons.

Polycystic ovary syndrome (PCOS) is characterized by the presence of at least 2 of 3 diagnostic criteria, including hyperandrogenism, menstrual dysfunction, and either multiple cyst-like follicles in the ovary or elevated anti-Müllerian hormone (AMH) (1). PCOS is reported to affect 6% to 20% of women of reproductive age worldwide (2, 3) and is the leading cause of anovulatory infertility (4, 5). Comorbidities of PCOS include obesity, insulin resistance, and impaired glucose tolerance, which can increase the risk of developing cardiovascular disease, hepatic steatosis, and type 2 diabetes (6). Hyperactive pulsatile secretion of centrally driven luteinizing hormone (LH) and reduced central sensitivity to gonadal hormone feedback is also a common feature of PCOS (7, 8), implicating a role for the brain in PCOS pathogenesis. Due to our poor understanding of the complex pathophysiology of PCOS, all treatment is symptom-based and there is no identified cure (1, 9).

Hyperandrogenism is evident in approximately 80% of women with PCOS (10). Excess androgen signaling in females likely contributes to the development of PCOS traits and may also serve as a potential therapeutic target. During pregnancy, individuals with PCOS have higher circulating androgen levels, and daughters of mothers with PCOS are more likely to develop PCOS features and associated comorbidities (11, 12). Likewise, androgen excess in critical developmental windows replicates PCOS-like traits in female mice, rats, sheep, and nonhuman primates (13). Conversely, treatment with androgen receptor (AR) blockers can restore ovulatory function (14) and gonadal steroid hormone feedback in PCOS patients (13) and restore estrous cyclicity in hyperandrogenic animal models of PCOS (15, 16).

Evidence from several androgen receptor knockout (ARKO) studies further emphasizes the importance of AR signaling in the pathogenesis of PCOS-like features. Global ARKO mice are protected from developing PCOS-like traits following chronic androgen exposure beginning at a peripubertal timepoint (17) or prenatal exposure to androgen excess (18). ARKO exclusively in the brain (17), the brain and adipose tissue (19), or in specific neuronal populations in the brain (20, 21) also provides some protection against the development of PCOS-like features in various hyperandrogenic PCOS-like models. Of interest, this protective effect is not evident in ovary-specific ARKO mice (17), supporting the importance of androgen signaling in the brain in the development of PCOS-like features.

One of the most dramatic and consistent features identified to date, particularly in prenatally androgenized models of PCOS is increased GABAergic innervation and transmission to the gonadotropin-releasing hormone (GnRH) neurons (22-27). These preclinical findings align with PCOS patient data indicating elevated cerebrospinal fluid levels of GABA (28, 29) and an altered response to positive allosteric modulators of GABA receptors (30). Although recognized as the major inhibitory neurotransmitter in the brain, GABA actions on GnRH neurons are largely excitatory via GABA_A_ receptors (31); this aligns with the hyperactive nature of the reproductive axis in PCOS (23). In mouse models, chronic activation of GABA neurons in the arcuate nucleus is sufficient to drive a PCOS-like phenotype (32), and the elevated GABAergic innervation and transmission to the GnRH neurons in PCOS-like mice can be reversed with anti-androgen treatment (15, 16).

The role of androgen actions specifically in GABA neurons, however, remains to be determined. This study investigates the hypothesis that androgen excess directly impacts the development of PCOS-like traits through GABA neurons. Using cre-lox transgenics, GABA-specific androgen receptor knockout (GABARKO) mice were generated and exposed to either acute prenatal androgen excess (PNA model) (16, 33) or chronic androgen excess from a peripubertal stage (peripubertal androgen or PPA model) (17, 34, 35) to determine whether AR signaling in GABA neurons is involved in the development of PCOS-like features.

## Methods

### Experimental Animals

Female mice were housed (3-5/cage) in a temperature and humidity-controlled environment with 12-hour light/dark cycles with ad libitum access to food and water at the University of Otago Biomedical Research Facility (Dunedin, New Zealand). All experimental procedures were approved by the University of Otago Animal Ethics Committee (Dunedin, New Zealand) and performed in accordance with the regulations of the Australasian and New Zealand Council for the Care of Animals in Research and Teaching.

### GABA Neuron–Specific ARKO

To generate mice with GABA neuron–specific knock out of AR (referred to as GABARKO), GABA vesicular transporter (VGAT)-ires-Cre (VGAT-Cre^+/-^) mice (36) were crossed with androgen receptor flox (AR^fl/fl^) mice (37). Experimental animals were produced by crossing heterozygous VGAT-Cre^+/-^; AR^fl/wt^ females with hemizygous AR^fl/Y^ males. To generate experimental animals with fluorescent GnRH neurons, VGAT-Cre^+/-^; AR^fl/wt^ females were bred with AR^fl/Y^; GnRH-GFP^+/+^ (38) males. VGAT-Cre^-/-^; AR^fl/fl^ females were used as controls (referred to as wild-type [WT]).

Cre-mediated excision of *Ar* exon 2 was confirmed by reverse-transcriptase polymerase chain reaction (RT-PCR). Briefly, 750 ng of total RNA extracted from mediobasal hypothalamic tissue (n = 4/group) was reverse transcribed using qScript (Quantabio, USA), and Touchdown PCR was carried out on 0.5 µL of cDNA, using a forward primer targeting Exon 1 of *Ar* (F:5′-GCCTCCGAAGTGTGGTATCC-3′), and a reverse primer targeting Exon 3 of *Ar* (R:5′-CCAGAGTCATCCCTGCTTCAT-3′). Loss of AR protein expression specifically in GABAergic neurons was confirmed by immunofluorescence (as described below) in mice crossed onto a tdTomato reporter line, generating VGAT-Cre:tdTomato (WT) and VGAT-Cre:tdTomato; AR^fl/fl^ (GABARKO) mice.

### Generation of PCOS-Like Mouse Models

Exposure to excess androgens, either chronically from a peripubertal timepoint or acutely during prenatal life, was used to generate model obese and lean PCOS-like models, respectively. Mice exposed to chronic dihydrotestosterone (DHT) from a peripubertal time point (PPA mice), modeling an obese PCOS-like phenotype, were generated as previously described (17, 34, 35). Briefly, at postnatal day (PND) 21, female mice were anesthetized with isofluorane (2%) and a small incision was made in the intrascapular region. A small silastic tube (1 cm in length, inner diameter: 1.47 mm; outer diameter: 1.95 mm, Dow Corning, Midland, MI, USA; catalog no. 508-006) containing 10 mg DHT or empty (Blank) and sealed with glue was then positioned subcutaneously. The incision was sutured to secure the implant for 13 weeks. The implants, made in-house, provide steady-state release of DHT for at least 6 months (39). During tissue collection, the presence of DHT powder within the implants was visually confirmed.

As previously described (16, 33), PNA mice mimicking lean PCOS were generated by injecting pregnant dams subcutaneously with 100 uL of either dihydrotestosterone (DHT; 250 μg) dissolved in sesame oil or sesame oil vehicle alone (VEH) on gestational days 16, 17, and 18. Female offspring of exposed dams were then studied from PND22.

### Experimental Design and Tissue Collection

**Figure 1.**
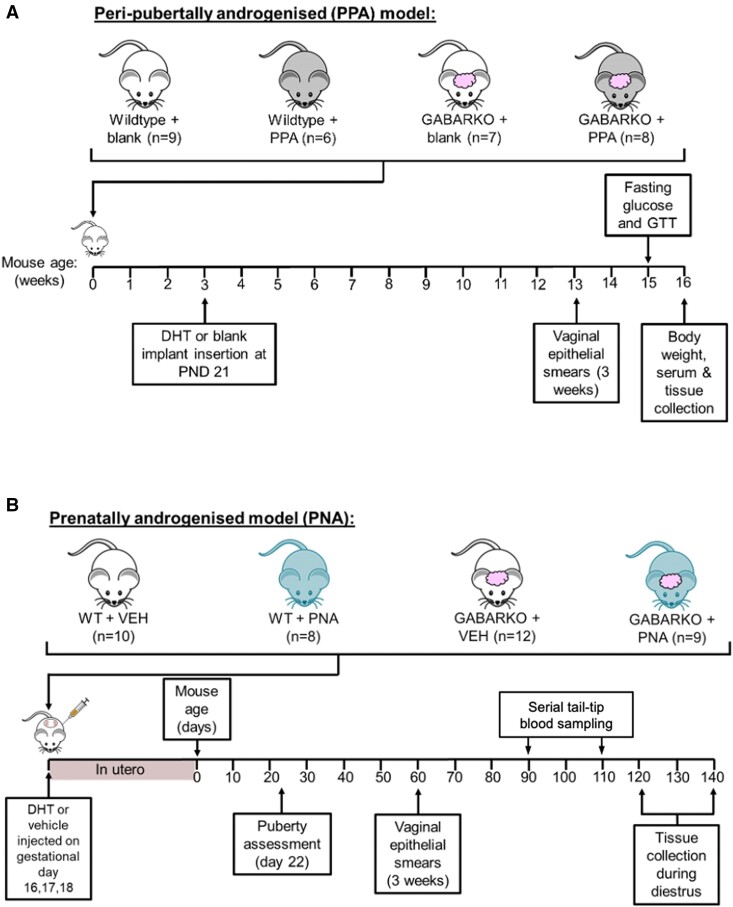
Experimental design. Two different hyperandrogenic models of PCOS and their respective controls were induced in both wild-type and GABA neuron–specific androgen receptor knockout (GABARKO) mice. A) Peripubertally androgenized (PPA) mice modeling both the reproductive and metabolic features of PCOS were generated by implanting a subcutaneous implant containing dihydrotestosterone (DHT) at postnatal day (PND) 21. Control mice had an empty (blank) implant inserted. Estrous cycling was assessed for 3 weeks following 10 weeks of chronic DHT exposure. Fasting glucose and glucose tolerance tests (GTT) were conducted at 15 weeks of age, before collection of tissues and serum at 16 weeks of age. B) Prenatally androgenized (PNA) mice mimicking lean PCOS were generated by injecting pregnant dams s.c. with 100 μL of either DHT (250 μg) dissolved in sesame oil or sesame oil vehicle alone (VEH) on gestational days 16, 17, and 18. Female offspring of exposed dams were then studied from postnatal day 25 for puberty onset, estrous cyclicity, and LH secretion.

A 2 × 2 factorial design was used to investigate the impact of GABARKO on the development of PCOS traits in 2 different PCOS-like models. PCOS models (PNA, PPA) and associated controls were generated in GABARKO and WT mice, resulting in 4 groups for each set of experiments (Fig. 1).

Treatment groups for the lean, PNA model included: WT + VEH (n = 10), WT + PNA (PCOS-like) (n = 8), GABARKO + VEH (n = 12) and GABARKO + PNA (PCOS-like) (n = 9). Puberty was assessed from PND22 and estrous cyclicity was assessed for 3 weeks from PND60. Between PND120 and PND140, females in the diestrus stage of the cycle were given a lethal overdose of sodium pentoparbital (60 mg/mL, 100 μL i.p.) and transcardially perfused with 4% paraformaldehyde (PFA; Sigma-Aldrich, MO, USA). Fixed brains were dissected out and cryoprotected in 30% sucrose-tris-buffered saline (TBS) at 4 °C. The brains were then cut into 4 series on a freezing microtome at a thickness of 30 μm. Free-floating brain sections were stored at −20 °C in cryoprotectant until used for immunohistochemistry.

Treatment groups for the obese, PPA model included: WT + blank (n = 9), WT + PPA (PCOS-like) (n = 6), GABARKO + blank (n = 7) and GABARKO + PPA (PCOS-like) (n = 8). Assessment of estrous cyclicity was carried out for 3 weeks, starting at 10 weeks postimplant through to the end of the 13-week endpoint. Glucose tolerance tests (GTT) were conducted after 12 weeks of PPA/blank implant exposure. At the end of the 13-week DHT exposure period, mice were euthanized by decapitation during the diestrus stage of the estrous cycle. Dissected tissues and a terminal blood sample for serum were collected and frozen.

### Assessment of Puberty Onset and Estrous Cyclicity

Puberty onset was measured in VEH control and PNA mice by daily examination for the age of vaginal opening. The day of first estrus was subsequently identified by daily vaginal smears.

In all groups, vaginal epithelial cell smears were collected daily for 21 consecutive days in adult mice to assess estrous cyclicity. Each smear was air-dried on a glass slide and stained with 0.1% Toluidine blue prior to analysis. Using a 10× objective, each stage of the estrous cycle was identified as follows; proestrus identified by the presence of round nucleated epithelial cells, estrus identified by the presence of anucleated keratinized epithelial cells, and diestrus identified by low numbers of cells mostly comprising of neutrophils (40). A complete estrous cycle was determined when the following stages of the estrous cycle had been visualized: proestrus, estrus, and diestrus.

### Fasting Blood Glucose and Glucose Tolerance Tests

In PPA groups, fasting blood glucose and GTTs were conducted during the twelfth week of exposure. First, mice were fasted for 6 hours (8:00 Am to 2:00 Pm) before a baseline blood glucose reading was taken using an Accu-Chek glucometer (Roche). Subsequently, the mice were injected intraperitoneally with glucose (2 g/kg body weight). Blood was drawn from a small incision in the tail and glucose was measured at 15-, 30-, 45-, 60-, 90-, and 120-minute intervals after glucose injection.

### Measuring Pulsatile Luteinizing Hormone

Mice were handled daily for habituation 4 weeks prior to serial tail-tip blood collection. As previously described (33, 41), 4 µL of whole blood was collected every 6 minutes for 2 hours (10:00 Am to 12:00 Pm) from a small incision in the tail tip. Blood was immediately suspended in 50 µL of phosphate-buffered saline (PBS) with 0.05% Tween-20 before being snap frozen on dry ice. Samples were stored at −20 °C until LH was measured by an ultrasensitive sandwich enzyme-linked immunosorbent assay (ELISA). Briefly, 96-well high affinity binding plates were coated with 50 µL/well of bovine LHb518B7 monoclonal antibody (1:1000 in PBS; RRID: AB_2756886; Pablo Ross, UC Davis, CA, USA) to serve as a capture antibody. A rabbit polyclonal antibody (1:10 000; AFP240590Rb; RRID: AB_2665533; National Hormone and Pituitary Program, Torrance, CA, USA) was used to detect bound LH in conjunction with a polyclonal-horseradish peroxidase conjugated goat anti-rabbit secondary antibody (1:1000; RRID: AB_2617138; DAKO). LH concentration was determined using a mouse LH-RP reference provided by Albert F. Parlow (National Hormone and Pituitary Program, Torrance, CA, USA). The sensitivity of this LH ELISA was 0.04 ng/mL, the intra- and inter-assay coefficients of variance were 5.2% and 14.4%, respectively. LH pulses were detected using the recently reformulated PULSAR software using G values optimized for detecting LH pulses in intact female mice, G1 = 3.5, G2 = 2.6, G3 = 1.9, G4 = 1.5, and G5 = 1.2. LH pulse amplitude for each pulse was computed within PULSAR for each pulse and basal LH was calculated as the average of the 10 lowest values (that were not zero values).

### Body and Fat Pad Weight and Adipocyte Morphology

Total body weight was determined immediately prior to euthanasia of the animal. For each mouse, the inguinal, parametrial, retroperitoneal, and brown fat pads were all individually weighed following dissection. Each fat pad was fixed in 4% PFA overnight at 4 °C and then stored in 70% ethanol. Parametrial and retroperitoneal fat pads from 4 mice from each treatment group were randomly selected and embedded in paraffin. The fat pads were sectioned at 8 μm. Three separate sections at least 160 μm apart were collected from each fat pad and stained with hematoxylin and eosin. Five images of each section were taken using an Olympus microscope (DP70) at 40×. Adipocyte area was analyzed and counted by an operator blinded to treatment group, using ImageJ version 1.53 software (NIH).

### Ovarian Morphology

Dissected ovaries from all animals were weighed, post-fixed in 4% PFA overnight and transferred to 70% ethanol for storage. Ovaries were processed through graded alcohols and either embedded in glycol methacrylate resin (Technovit 7100; Heracus Kulzer) or paraffin prior to sectioning. Paraffin-embedded ovaries from PNA groups were sectioned at 5 μm thickness, collecting every tenth section to allow for stereological analysis of the whole ovary and subsequently stained with hematoxylin and eosin. All sections were imaged at 4× on an Olympus BX51 microscope. Resin-embedded ovaries from PPA groups were serially sectioned at 20 μm, stained with periodic acid Schiff, and counterstained with hematoxylin. Every third section was imaged at 4× on an Olympus DP70 microscope. The photos were analyzed by an operator who was blinded to treatment group. Preovulatory follicles were counted where the presence of the ova was evident, and corpora lutea were counted where a core was visible. For each animal, a 20× image containing the largest preovulatory follicle was used to analyze the theca cell, and granulosa cell composition of preovulatory follicles. These were calculated as a percentage of the total follicle using QuPath (42).

### Immunofluorescence

To assess AR protein expression in GABA neurons, brain sections from VGAT-Cre:tdTomato (WT) and VGAT-Cre:tdTomato; AR^fl/fl^ (GABARKO) mice underwent free-floating immunohistochemistry labeling for AR (n = 3/group). GABAergic neurons were identified by endogenous Cre-dependent fluorescent tdTomato reporter expression. Every fourth coronal brain section was washed thoroughly in TBS, then permeabilized in a 0.3% Triton-X TBS solution for 10 minutes. Sections were then incubated with 10% fetal bovine serum in incubation solution (TBS with 0.3% Triton X-100 and 0.25% bovine serum albumin) for 1 hour, before being washed in TBS and finally blocked in a 5% normal goat serum (NGS) solution for 1 hour. Sections were incubated with a rabbit anti-AR primary antibody (1:200, Abcam AB105225) in incubation solution containing 2% NGS for 72 hours at 4 °C. Sections were washed again in TBS then incubated in goat anti-rabbit AlexaFluor647 (1:500, Thermo Fisher AlexaFluor A21244) to visualize AR immunolabeling. Primary antibody omission resulted in a complete absence of fluorescent labeling in the far-red spectrum used to detect the 647 fluorophore.

To assess GnRH neuron morphology and presynaptic GABAergic inputs, rostral preoptic area brain sections from PNA groups underwent free-floating immunohistochemistry labeling for VGAT and GFP as previously reported (15, 25). Briefly, every fourth serial section was washed thoroughly in TBS, blocked with 5% NGS in incubation solution for 60 minutes and then incubated in polyclonal rabbit anti-VGAT primary antibody (1:750; RRID: AB_887869; Synaptic Systems) and polyclonal chicken anti-GFP (1:3000; RRID: AB_2307313; Aves Labs Inc.) for 72 hours at 4 °C. VGAT staining was detected using goat anti-rabbit AlexaFluor647 (1:500; A21244; Thermo Fisher AlexaFluor), while GFP was visualized with goat anti-chicken AlexaFluor488 (1:500; RRID: AB_142924; Molecular Probes, Invitrogen). Primary antibody omission served as a negative control.

### Confocal Microscopy and Analysis

GnRH neurons (8-10/animal) were randomly selected across 2 representative rostral preoptic area sections from each animal and images were captured on a Nikon A1R confocal microscope (Nikon Instruments Inc., Tokyo, Japan), with 488 and 647 nm lasers at 40× magnification (0.5 μm Z-step, 1 AU pinhole, digital magnification 2×). Using Nikon NIS-Elements AR 4.5 (Nikon Instruments Inc.) software, images were analyzed by a researcher blinded to treatment group. VGAT apposition and GnRH spine density were quantified for each GnRH neuron soma and primary dendrite as previously described (15, 25).

### Statistical Analysis

Statistical analysis was performed using GraphPad Prism 9. All data were tested for normality using the D’Augusto and Pearson normality omnibus test. Most statistical differences were tested using 2-way ANOVA to assess the effect of genotype (G), treatment (T) and genotype X treatment interaction (TxG) with post hoc test using Tukey multiple-comparison test, corrected for multiple comparisons. Validation of AR removal from GABA neurons was assessed using an unpaired Student *t* test. *P* values < .05 were considered statistically significant.

## Results

### Confirmation of Androgen Receptor Knockout

**Figure 2.**
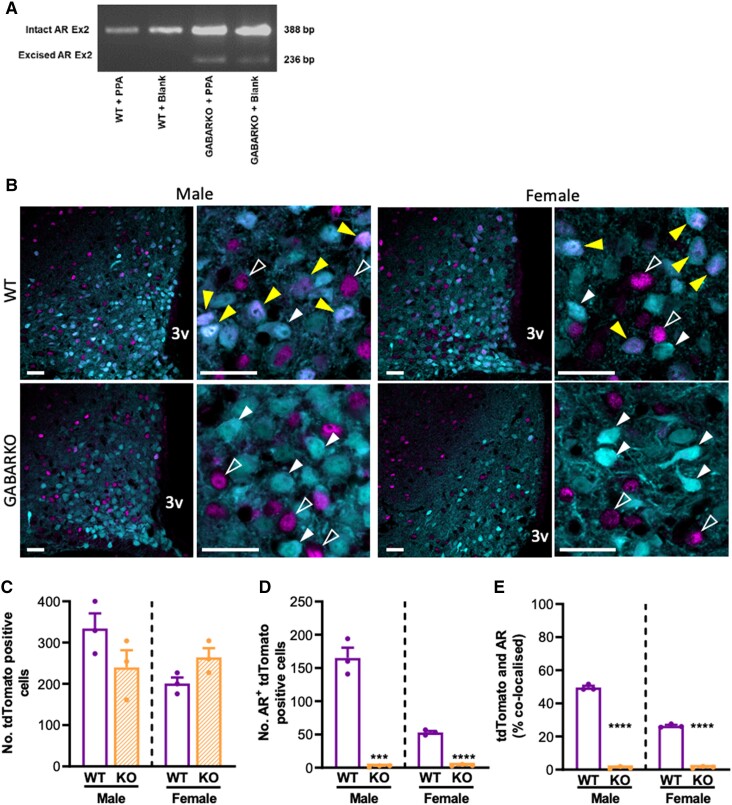
Confirmation of transgenic androgen receptor knockout in GABA neurons. A) RT-PCR confirmation of Cre-mediated excision of Androgen receptor (AR) exon 2 in brain tissue from GABARKO mice. The intact AR exon 2 PCR product (388 bp) was found across all groups, denoting the intact AR gene in non-GABA neurons, while the excised AR exon 2 PCR product (236 bp) was only identified in GABARKO animals. B) Representative single confocal stack images of the medial arcuate nucleus from male and female VGAT-Cre:tdTomato (WT) or VGAT-Cre:tdTomato:ARfl/fl (KO) mice showing androgen receptor (magenta) and GABA neurons indicated by VGAT-Cre-dependent tdTomato expression (cyan). In higher magnification images, yellow arrows indicate androgen receptor and GABA neuron colocalization, empty white arrows indicate single label AR neurons and filled white arrows indicate single label GABA neurons. C) Mean ± SEM number of tdTomato positive cells in male and female WT (open purple bars) and KO mice (shaded orange bars). D) Mean ± SEM number of tdTomato-expressing cells colocalized with AR in male and female WT and KO mice. E) Mean ± SEM proportion of AR positive tdTomato-expressing neurons in male and female WT and KO mice. n = 3 for all groups, unpaired Student *t* test. *** *P* < .001, **** *P* < .0001. Scale bar = 30 μm. Abbreviation: 3v, third ventricle.

RT-PCR on brain tissue from all PPA mice and blank controls, using specifically designed *Ar* exon 2 primers, demonstrated the excision of the *Ar* band in tissue from all GABARKO mice. As expected, the intact 388 bp exon 2 PCR product was present in samples from every mouse tested due to heterogeneous cell types in the brain. An additional 236 bp band was present exclusively in GABARKO mice, indicating an excised *Ar* exon 2 in the brain (Fig. 2A).

Immunofluorescent labeling of AR in brain slices from adult male and female VGAT-Cre:td-Tomato (WT) and VGAT-Cre:tdTomato; AR^fl/fl^ (GABARKO) mice confirmed the loss of AR specifically in GABA neurons in this transgenic cross (Fig. 2B). The number of tdTomato positive cells (VGAT reporting of GABAergic neurons) was not different between males and females, nor impacted by ARKO (Fig. 2C). Approximately 50% of GABAergic neurons were colocalized with AR in males and approximately 30% of GABAergic neurons were colocalized with AR in females (Fig. 2D). The number and proportion of GABAergic neurons co-expressing AR was almost completely abolished, with fewer than 1% of GABAergic neurons colocalized with AR in both male and female GABARKO mice (Fig. 2D and 2E, *P* < .0001).

### Androgen Signaling in GABA Neurons Is Not Required for the Development of Reproductive Features in a Chronic DHT Model of PCOS

**Figure 3.**
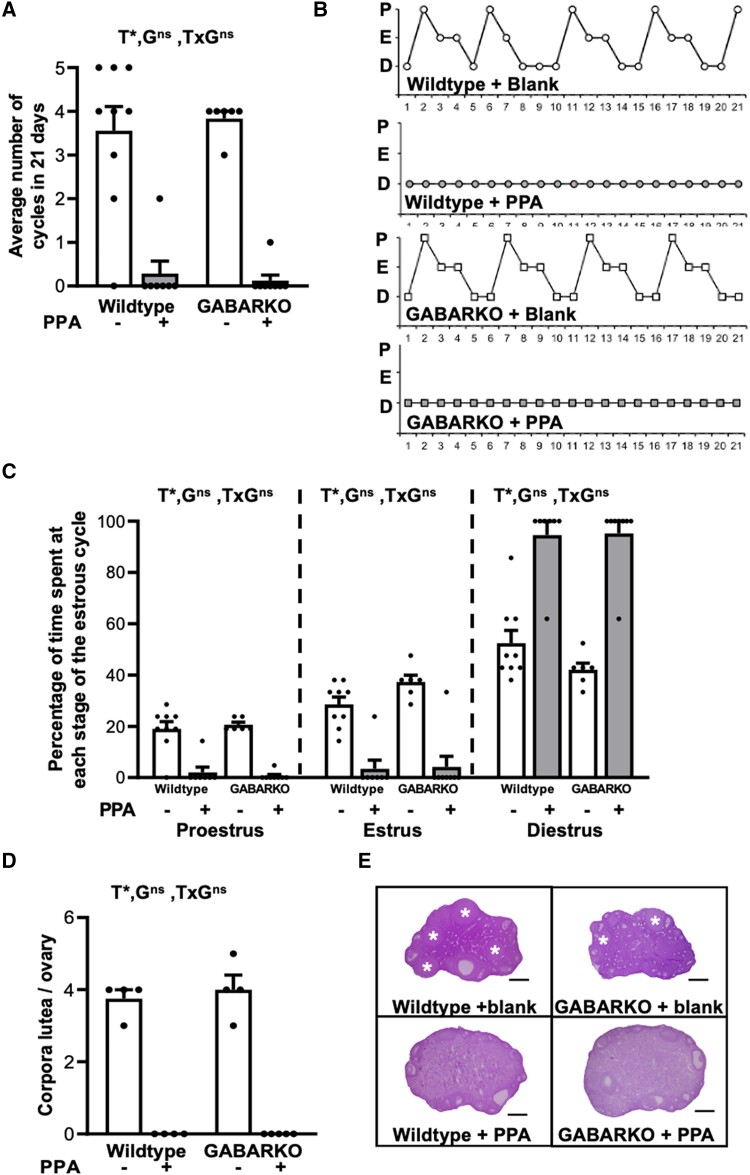
PPA Estrous cycling and ovarian morphology. (A) Number of completed cycles in a 21-day period, showing DHT (Dihydrotestosterone) induced acyclicity (DHT main effect *P* < .0001), but no genotype specific effect on cycling, n = 6-9 mice per experimental group. (B) Representative estrous cycle patterns in female mice. Abbreviations: E, estrus, and D, diestrus; P, proestrus. (C) Percentage of time spent at each stage of the estrous cycle, showing disrupted estrous cycling in DHT- exposed female mice (DHT main effect *P* < .0001). (D) Number of corpora lutea per ovary shows that DHT exposure induced anovulation (DHT main effect *P* < .0001) but there was no difference between the genotypes, n = 4-5 ovaries per experimental group. (E) Representative histological cross-sections of ovaries from each experimental group. Stars denote corpora lutea. Abbreviations: G, genotype; T, DHT treatment; ns, no significant difference; *, significant main effect difference. Data are expressed as the mean ± SEM, 2-way ANOVA. Scale bar is 500 μm.

Both WT + blank and GABARKO + blank mice exhibited normal estrous cyclicity, with an average of ∼ 3.5 cycles over 21 days (Fig. 3A and 3B). Conversely, DHT exposure caused a significant main effect on estrous cyclicity (*P* < .0001) as the majority of WT + PPA mice were acyclic (Fig. 3A and B) and remained in a diestrus state (main effect of DHT, *P* < .0001), characterized by the strong presence of leukocytes in vaginal smears for the majority of the estrous cycle (Fig. 3B and 3C). There was no difference in cyclicity observed between GABARKO and WT PPA mice, as both groups remained acyclic (Fig. 3B and C).

DHT exposure had a significant main effect (*P* < .0001) on ovarian corpora lutea numbers, as corpora lutea were absent from all WT + PPA ovaries (Fig. 3D and 3E). There was no difference in corpora lutea number between GABARKO and WT + PPA mice, suggesting that AR signaling in GABA neurons is not implicated in the development of reproductive PCOS-like features in this mouse model (Fig. 3A-E).

### Androgen Signaling in GABA Neurons Is Not Required for the Development of Metabolic Traits in a Chronic DHT Model of PCOS

**Figure 4.**
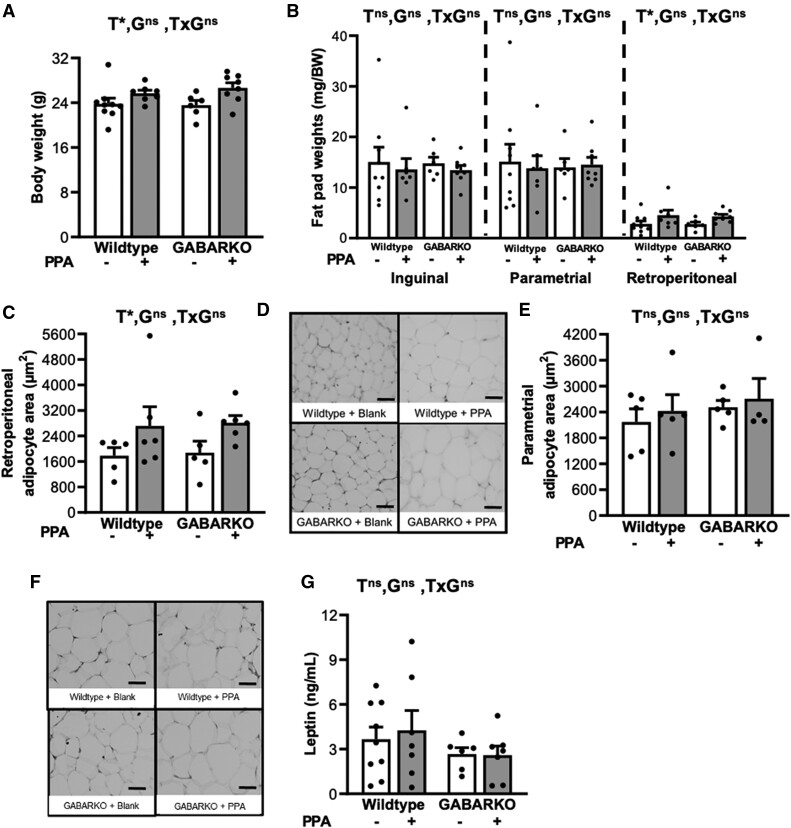
Body weight, fat pad weights, adipocyte, and leptin. (A) Body weight shows a significant main effect of DHT (Dihydrotestosterone) treatment (*P* < .05), but no effect of genotype. (B) Inguinal, parametrial, and retroperitoneal fat pad weights. There was no effect of DHT treatment or genotype on inguinal or parametrial fat pad weights. Retroperitoneal fat pad weight showed a main effect of DHT exposure (*P* < .05), but there was no effect of genotype. (C) Adipocyte size in retroperitoneal fat pads showing a significant main effect of DHT on adipocyte hypertrophy (*P* < .05), but no effect of genotype. (D) Representative histological sections of retroperitoneal fat pads of mice from each experimental group showing the increase in adipocyte area in DHT-exposed mice. (E) Parametrial adipocyte area showing no difference between any of the treatment groups. (F) Representative histological sections of parametrial fat pads of mice from each experimental group showing no difference between treatment groups. (G) Serum levels of leptin showing no significant difference in circulating leptin levels between any of the experimental groups. For A, B, and G, n = 6-9 mice per experimental group; for C and E, n = 5-6 mice per experimental group. For all graphs, abbreviations: G, genotype; T, DHT treatment; ns, no significant difference; *, significant main effect difference. Data are expressed as the mean ± SEM, two-way ANOVA. Scale bar is 50 μm.

To assess the effect of androgen signaling in GABA neurons on PCOS-like metabolic features, body weight and adipocyte histology were assessed. As expected, there was a significant main effect of DHT exposure on body weight, where WT + PPA mice displayed a significantly higher total body weight compared to WT + blank mice (*P* < .05) (Fig. 4A). There was no significant difference in body weight found between GABARKO + PPA and WT + PPA mice (Fig. 4A). A similar main effect was observed in the retroperitoneal fat pad weights, with exposure to DHT associated with a significant increase in weight compared to blank controls in wild-type and GABARKO mice (*P* < .05) (Fig. 4B). There was no significant difference in retroperitoneal fat pad weight between genotypes and there was no significant difference in inguinal or parametrial fat pad weight between any of the treatment groups. Histological analysis of the parametrial and retroperitoneal adipocytes revealed a main effect of DHT exposure in the retroperitoneal fat pads as WT + PPA mice and GABARKO + PPA mice displayed a significant increase (*P* < .05) in adipocyte area compared to control mice (Fig. 4C and 4D). GABARKO did not affect retroperitoneal adipocyte size. No significant difference in parametrial adipocyte area was observed between any of the treatment groups (Fig. 4E and 4F). Additionally, average circulating levels of leptin, an adipocyte derived hormone, were not different between any of the 4 treatment groups (Fig. 4G).

**Figure 5.**
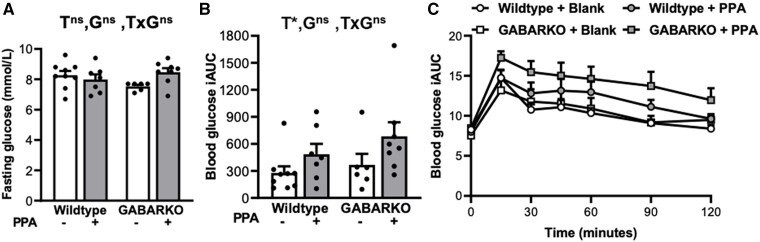
Fasting glucose and glucose tolerance test (GTT). (A) Fasting glucose levels showing no significant difference in fasting glucose levels between any of the treatment groups. (B) Mean incremental area under the curve analysis of GTT showing a main effect of DHT (Dihydrotestosterone) treatment (*P* < .05) but no effect of genotype. (C) Mean blood glucose curves following GTT. For all graphs, n = 6-9 per experimental group and data are expressed as the mean ± SEM, 2-way ANOVA. Abbreviations: G, genotype; T, DHT treatment; ns, no significant difference; *, significant main effect difference.

Fasting blood glucose and blood glucose incremental area under the curve (iAUC) were assessed to determine overall glucose homeostasis. The fasting glucose measurements revealed no significant differences between any of the treatment groups (Fig. 5A). Androgen exposure had a main effect on the blood glucose iAUC measurements (*P* < .05); however, there was no significant difference between the genotypes (Fig. 5B and 5C), suggesting that androgen signaling in GABA neurons is not required for the impaired glucose homeostasis of this hyperandrogenic PCOS-like model.

### GABARKO Does Not Rescue Prenatal Androgen–Induced Impairments in Estrous Cyclicity or Impact Ovarian Morphology

**Figure 6.**
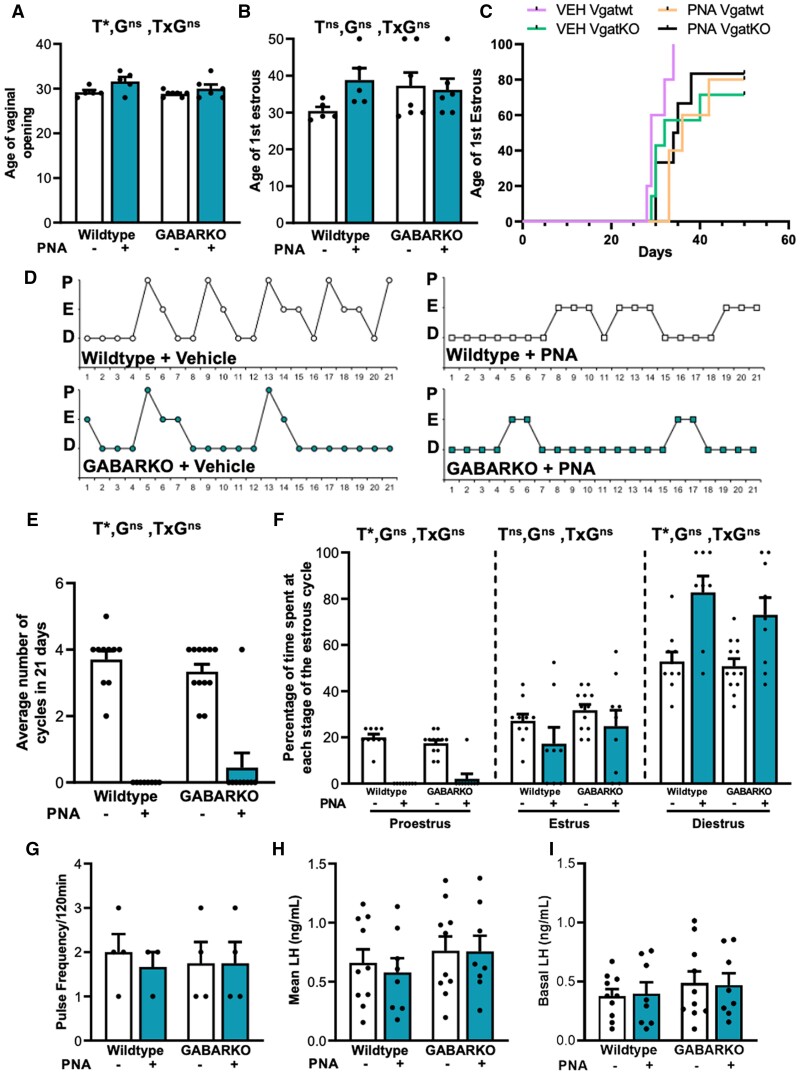
PNA estrous cycling. **(**A) Age of vaginal opening showing a main effect of treatment (*P* < .05). (B) Age of first estrous with no significant difference between any of the treatment groups. (C) A cumulative survival plot showing the timing of first estrous for each treatment group. (D) Representative estrous cycle patterns in female mice. P, proestrus; E, estrus, and D, diestrus. (E) Average number of completed cycles in 21 days showing a main effect of androgen exposure (*P* < .0001). (F) Percentage of time spent at each stage of the estrous cycle, showing an androgen-mediated main effect on estrous cycling disruption (*P* < .0001). (G) Mean LH frequency in animals with detectable LH pulses. Mean (H) and basal (I) LH concentration across all animals. n = 5-12 mice per experimental group. Abbreviations: G, genotype; T, PNA treatment; ns, no significant difference; *, significant main effect difference. Data are expressed as the mean ± SEM, 2-way ANOVA.

Androgen exposure led to a delay in the age of vaginal opening in PNA mice compared to non-PNA mice (DHT main effect, *P* < .05) (Fig. 6A). The mean day of first estrous smear were not different between groups (Fig. 6B). However, while 100% of the WT + VEH animals reached first estrous day by 34 days of age, all other groups included animals that did not reach first estrous day by the completion of the observation period (21 days) (Fig. 6C). The majority of PNA mice were acyclic (DHT main effect, *P* < .0001) and this was not impacted by genotype (Fig. 6D and 6E). All but one GABARKO + PNA mouse had absent cycles (Fig. 6E). Both WT + PNA and GABARKO + PNA animals spent a greater amount of time in diestrus, and a reduced amount of time spent in proestrus when compared to WT + VEH and GABARKO + VEH mice (main effect, both *P* < .0001) (Fig. 6F).

LH secretion was measured through serial tail-tip blood sampling from gonadally intact female mice at diestrus. Characteristic LH pulses were not consistently detected across all animals; however, in those animals in which pulses were detected (Supplementary Fig. S2) (43), the LH pulse frequency, amplitude, and area under the curve (AUC) were not different between groups (Fig. 6G, Supplementary Fig. S2A and S2B) (43). Mean and basal LH, measured across animals with and without detectable pulses, was also not different between groups (Fig. 6H and 6I).

**Figure 7.**
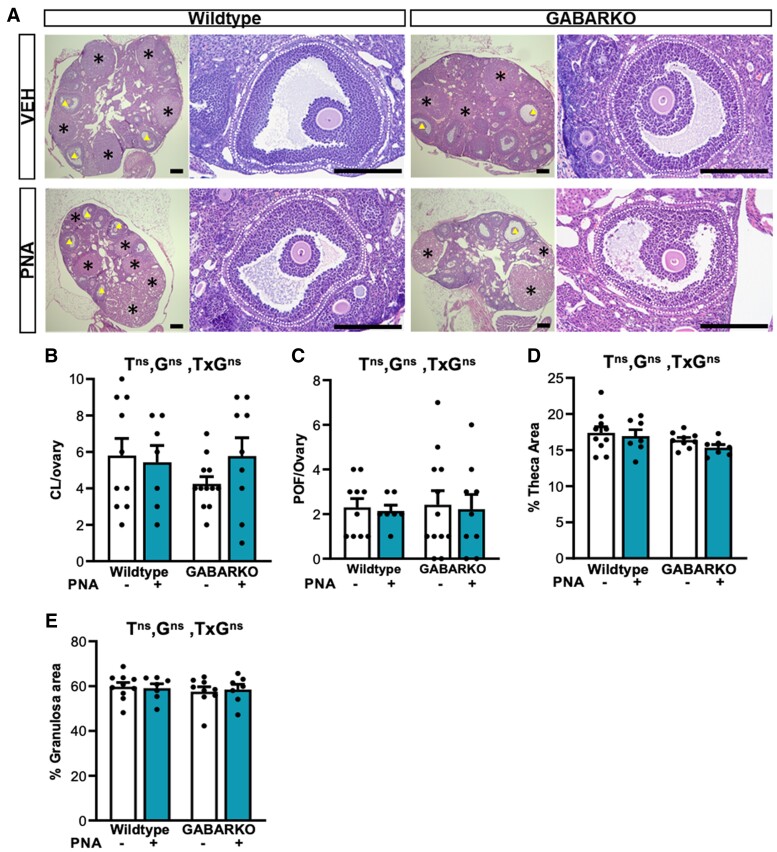
PNA histological assessment of ovarian morphology and preovulatory follicle structure. (A) Representative images of ovaries and preovulatory follicles. (B) Number of corpora lutea per ovary showing no significant differences in CL number per ovary between any of the experimental groups. (C) Number of preovulatory follicles per ovary showing no significant differences between the treatment groups. (D) Percentage theca area showing that the experimental groups are not significantly different from one another. (E) Percentage granulosa area showing no significant difference between any of the experimental groups. n = 8-12 mice per experimental group. Abbreviations: G, genotype; T, PNA treatment; ns, no significant difference; *, significant main effect difference. Data are expressed as the mean ± SEM, 2-way ANOVA.

Despite the acyclicity evident in PNA females, there were no significant differences evident in morphological measurements of ovarian structures between groups. The average number of corpora lutea and preovulatory follicles was similar between groups (Fig. 7A-7C). The proportion of preovulatory follicles occupied by theca and granulosa cell layers were also not significantly different between groups (Fig. 7A, 7D, and 7E).

### GABARKO Rescues Prenatal Androgen–Induced Plasticity in GABAergic Innervation to the GnRH Neurons

**Figure 8.**
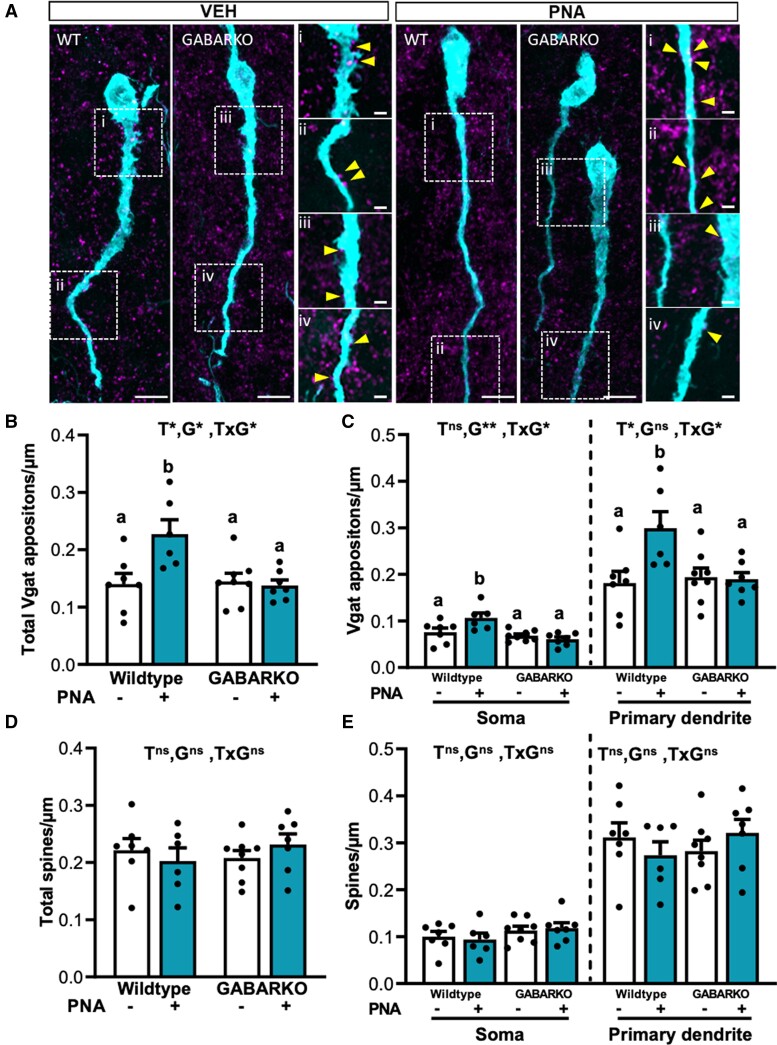
PNA-induced elevation in VGAT contact to GnRH neurons is dependent upon AR expression in GABA neurons. (A) Representative maximum projection confocal images of GnRH neurons (cyan) and VGAT puncta (magenta), with cropped images highlighting GnRH neurons spines (closed arrowheads). (B) Mean ± SEM of VGAT puncta per micron closely apposing the GnRH neuron cell body for the whole neuron showing main effects of treatment, genotype and an interactive effect (All *P* < .05). (C) VGAT puncta per micron closely apposing the GnRH neuron from the soma showing main effect of genotype (*P* < .01) and an interaction (*P* < .05), and from the primary dendrite showing a main effect of treatment (*P* < .05) and an interactive effect (*P* < .05). (D) Mean ± SEM of GnRH neuron spines per micron for the whole neuron showing no significant effect. (E) GnRH neuron spines per micron for the soma and the primary dendrite showing no significant difference between any of the treatment groups. n = 7 for VEH WT, PNA KO, n = 6 for PNA WT, n = 8 for VEH KO. Abbreviations: T, PNA treatment; G, genotype; ns, no significant difference; *, significant main effect difference. Data are expressed as the mean ± SEM, 2-way ANOVA. Different letters above each bar denote significant statistical differences as assessed via Tukey post hoc test. Scale bar = 8 μm, inserts = 2 μm.

GnRH neuron spine density and the density of immunoreactive VGAT appositions to GnRH neurons was measured within specific neuronal compartments, including the soma and within 15 µm lengths of the primary dendrite (Fig. 8). No significant interactions were identified for total or compartmental GnRH neuron spine density between groups (Fig. 8D and 8E).

There was, however, a significant difference in the mean density of immunoreactive VGAT appositions to GnRH neurons between groups (Fig. 8B and 8C). There was a significant interaction between treatment and genotype for total density of VGAT appositions (main effect, *P* = .0107) (Fig. 8B). In VEH-treated animals, GABARKO did not significantly impact the mean VGAT apposition density to GnRH neurons. As expected, a post hoc Tukey test revealed WT + PNA females had a significant increase in VGAT apposition density to GnRH neurons compared to WT + VEH females (*P* = .0093) (Fig. 8B). GABARKO significantly decreased the total VGAT apposition density to GnRH neurons in PNA animals to levels that were not different to VEH controls (Tukey post hoc, *P* = .4622). This pattern of changes in total VGAT apposition density reflected changes in apposition density at the soma and the primary dendrite (Fig. 8C). There was a significant interaction between treatment and genotype (*P* = .0144), with a genotype main effect impacting VGAT apposition density at the soma of GnRH neurons (*P* = .0012) and the primary dendrite (*P* = .0175). Somatic VGAT apposition density was significantly lower in GABARKO + PNA mice compared to WT + PNA females (Tukey post hoc, *P* = .0013) and not different to WT or GABARKO VEH groups. Likewise, elevated putative GABAergic inputs to the primary dendrite in WT + PNA animals was significantly lower in GABARKO + PNA females (Tukey post hoc, *P* = .0225) and not different to VEH groups. An assessment of inputs along the primary dendrite length showed significantly increased VGAT appositions in WT + PNA mice compared to all other groups between 15 and 30 μm of the proximal primary dendrite (Tukey post hoc, *P* = .0318). (Supplementary Fig. S1) (43).

## Discussion

Androgen excess is both a hallmark feature of PCOS and a likely culprit in driving the development of PCOS-related reproductive and metabolic neuroendocrine disturbances. Identifying the specific targets of androgen-mediated dysfunction will ultimately aid our understanding of PCOS pathogenesis and the identification of novel treatment pathways. To assess whether GABA neurons are direct and critical targets of androgen signaling, a transgenic approach was used to knockout androgen receptors from all GABAergic neurons in 2 well-characterized mouse models of PCOS. We identified that the PCOS-like reproductive and metabolic traits in PPA mice and the PCOS-like reproductive traits in PNA mice were not significantly altered or improved in GABARKO mice with androgen signaling specifically blocked in GABA neurons. We did, however, find that GABARKO abolished the PNA-induced elevation in putative GABAergic synapses to GnRH neurons, a well-documented plastic feature of PNA mice. Together, these data suggest that AR signaling in GABA neurons is unlikely to play a direct and critical role in the development of PCOS-like reproductive and metabolic dysfunction but highlight the importance of direct androgen actions in GABA neurons in the development of the GnRH neuronal network.

Female androgen excess can manifest in reproductive, endocrine, and metabolic dysfunction that resembles PCOS in several different mammalian species (13). Consistent with previous studies, both PPA and PNA mice in the present study were acyclic, indicating ovulatory dysfunction (17, 18, 44). Additionally, PPA mice displayed an increase in total body weight, retroperitoneal fat pad weight, and impaired glucose tolerance, indicating metabolic dysfunction, as reported previously (44). In this study, the manifestation of PNA reproductive traits was less prominent than has been previously reported (15, 25, 33, 44). The PNA mice in this study were clearly and consistently acyclic, but ovarian morphology was not different to non-androgenized mice. In addition, serial tail-tip blood sampling for LH did not detect elevated LH pulse frequency as reported previously (25). These discrepancies may reflect unidentified drift in the model, genetic background, and/or technical limitations. Detection of LH pulses in gonadally intact animals is particularly challenging (45), suggesting that technical limitations may have contributed to this result. The mismatch between estrous cyclicity and ovarian morphology is more difficult to interpret. In any case, PNA-induced acyclicity is not rescued in PNA GABARKO mice, suggesting that PNA is acting through another target.

Cre-lox transgenics allowed us to eliminate AR signaling exclusively in all VGAT-expressing GABA neurons. The AR gene was successfully excised in GABARKO mice, resulting in a complete absence of detectable AR protein in VGAT-expressing GABA neurons. Despite characterizing the complete loss of AR in GABA neurons, no robust reproductive or metabolic phenotype was evident in vehicle-treated GABARKO mice, indicating that AR actions in GABA neurons are not required for normal function. Likewise, GABARKO did not protect androgenized females from developing reproductive and metabolic dysfunction. While these data indicate that GABA AR is not essential, the lack of a robust phenotype could reflect compensation following the loss of androgen sensitivity in GABA neurons from an early developmental timepoint. Determining whether loss of the AR gene in adults following normal development results in a similar lack of phenotype will be important to rule out developmental compensation.

The rationale for investigating AR in the brain and specifically in GABA neurons comes from evidence indicating that brain AR is necessary for PPA to evoke reproductive and metabolic features (17), and a large body of evidence linking PNA and the development of PCOS-like features with modified GABA regulation of GnRH neurons (22-27). PNA in females, induced by testosterone, DHT, or AMH, evokes an early and persistent increase in GABA contact with and transmission to GnRH neurons (15, 24, 25, 27, 46) that, in some cases, is associated with increased LH pulsatility (25, 46). While PPA drives reproductive and metabolic impairments that resemble PCOS (44), it does not model the impaired gonadal steroid hormone feedback and hyperactive GnRH/LH secretion of PNA models or the elevated GABAergic innervation to GnRH neurons (35). Therefore, the absence of any phenotype rescue in the PPA GABARKO animals may not be that surprising. However, it does rule out GABA neurons in general as the critical direct central targets of androgen excess, implicating glutamatergic populations as more likely targets.

Cell type–specific AR knockout studies in other PCOS-like models indicate the importance of the largely glutamatergic KNDy neurons (co-expressing kisspeptin, neurokinin B, and dynorphin) in androgen excess–mediated dysfunction. KNDy neurons are important mediators of gonadal steroid hormone feedback (47) and drive the pulsatile secretion of GnRH/LH (48). Kisspeptin-specific androgen receptor knockout (KARKO) protects mice from developing most PCOS-like features. This has been reported in both the letrozole-treated hyperandrogenic model of PCOS (20) and in the prenatal AMH-treated model of PCOS (21). It remains to be determined whether KARKO is also protective of the features developed by PPA and PNA. A neurokinin B receptor antagonist was found to rescue some of the metabolic PCOS traits in the PPA model (49), suggesting that at least some aspects of hyperandrogenic dysfunction are occurring through KNDy neurons in this model. Although no changes in kisspeptin excitability are evident in the PNA model (50), PNA does modify the gene expression and innervation of KNDy neurons (51). Other potential direct androgen targets include the AgRP/NPY neurons, well known for their regulation of feeding and energy homeostasis (52) and also important in regulating the reproductive axis (53, 54). AgRP/NPY neurons express AR (55) and appear to be plastic in some PNA models (56).

GABARKO, while not effective in blocking the development of PCOS-like pathology, clearly blocked elevated GABAergic input to GnRH neurons in PNA mice. These data raise several points of discussion. First, this blockade of a known PNA outcome suggests that AR excision occurred prior to DHT exposure in this transgenic cross. This suggests that we are blocking the programming effects of maternal androgen excess through GABA neurons in the PNA GABARKO mice. Secondly, a rescue of the GABA input phenotype without rescue of acyclicity suggests that elevated GABA innervation to GnRH neurons is likely not the primary mechanism by which PNA evokes acyclicity. PNA programming of other central and peripheral targets is sufficient to maintain impaired reproductive cycles. Lastly, these data suggest that the PNA-induced plasticity in GABA innervation to the GnRH neurons requires direct androgen actions in GABA neurons. Evidence suggests that PNA is associated with reduced microglia pruning of GABA terminals during development (57). The present data would suggest that this mechanism is initiated through the GABA neurons. While evidence supports that GABA neurons in the arcuate nucleus contribute to the majority of synaptic plasticity in PNA, we cannot rule out the importance of other GABA populations. In late gestation, only a small proportion of arcuate nucleus GABA neurons express AR, while GABA cells in the ventral lateral septum and medial preoptic area show a higher co-expression (58).

While the present data suggest that GABA neurons are not the critical direct targets of androgen excess, they do not diminish the potential importance of altered GABA tone in the development of PCOS features. Clinically, elevated GABA tone is evident in PCOS patients (28, 29) and PCOS patients respond differently to the GABA A modulator allopregnenalone (30). Both clinical and preclinical evidence suggests that pharmacologically or chemogenetically elevating GABA tone can evoke PCOS-like dysfunction (32, 59). Therefore, the relationship between elevated testosterone and GABA signaling is complex and bidirectional.

Together, these data suggest that while critical, direct androgen actions mediating PCOS pathogenesis are likely to involve other cell phenotypes, the AR-mediated programming of GABA input to GnRH neurons is dependent upon direct androgen actions in GABA neurons. Future studies will be important in identifying and determining the role of other neuronal and non-neuronal targets involved in PCOS pathogenesis and exploring the mechanisms underpinning AR-mediated plasticity of developmental brain wiring.

## Data Availability

The data supporting the findings of this study are available on request from the corresponding author.

## Abbreviations

AMH: anti-Müllerian hormone
AR: androgen receptor
ARKO: androgen receptor knockout
DHT: dihydrotestosterone
ELISA: enzyme-linked immunosorbent assay
GABARKO: GABA-specific androgen receptor knockout
GnRH: gonadotropin-releasing hormone
GTT: glucose tolerance test
iAUC: incremental area under the curve
KNDy: kisspeptin/neurokinin B/dynorphin expressing
LH: luteinizing hormone
NGS: normal goat serum
PBS: phosphate-buffered saline
PCOS: polycystic ovary syndrome
PFA: paraformaldehyde
PNA: prenatal androgen excess
PND: postnatal day
PPA: peripubertal androgen excess
RT-PCR: reverse-transcriptase polymerase chain reaction
TBS: Tris-buffered saline
VEH: vehicle
WT: wild-type
